# Sparstolonin B Reduces Estrogen-Dependent Proliferation in Cancer Cells: Possible Role of Ceramide and PI3K/AKT/mTOR Inhibition

**DOI:** 10.3390/ph17121564

**Published:** 2024-11-21

**Authors:** Yağmur Dilber, Hanife Tuğçe Çeker, Aleyna Öztüzün, Bürke Çırçırlı, Esma Kırımlıoğlu, Zerrin Barut, Mutay Aslan

**Affiliations:** 1 Department of Medical Biochemistry, Faculty of Medicine, Akdeniz University, Antalya 07070, Turkey; yagmursarica88@gmail.com (Y.D.); tugceker159@gmail.com (H.T.Ç.); aleyna_042@hotmail.com (A.Ö.); 2 Department of Medical Biotechnology, Institute of Health Sciences, Akdeniz University, Antalya 07070, Turkey; burkecircirli@outlook.com; 3 Department of Histology and Embryology, Faculty of Medicine, Akdeniz University, Antalya 07070, Turkey; esmakirimlioglu@gmail.com; 4 Faculty of Dentistry, Antalya Bilim University, Antalya 07070, Turkey; zerrin.barut@antalya.edu.tr

**Keywords:** Sparstolonin B, ceramide, apoptosis, PI3K, AKT, mTOR

## Abstract

**Background:** The aim of this study was to determine the effect of Sparstolonin B (SsnB) on cell proliferation and apoptosis in human breast cancer (MCF-7) and human ovarian epithelial cancer (OVCAR-3) cell lines in the presence and absence of estradiol hemihydrate (ES). Phosphoinositol-3 kinase (PI3K), phosphorylated protein kinase B alpha (p-AKT), phosphorylated mTOR (mechanistic target of rapamycin) signaling proteins, and sphingomyelin/ceramide metabolites were also measured within the scope of the study. **Methods:** The anti-proliferative effects of SsnB therapy were evaluated over a range of times and concentrations. Cell proliferation was determined by measuring the Proliferating Cell Nuclear Antigen (PCNA). PCNA was quantified by ELISA and cell distribution was assessed by immunofluorescence microscopy. MTT analysis was used to test the vitality of the cells, while LC-MS/MS was used to analyze the amounts of ceramides (CERs), sphingosine-1-phosphate (S1P), and sphingomyelins (SMs). TUNEL labeling was used to assess apoptosis, while immunofluorescence staining and enzyme-linked immunosorbent assay (ELISA) were used to measure the levels of PI3K, p-AKT, and p-mTOR proteins. **Results:** Sparstolonin B administration significantly decreased cell viability in MCF-7 and OVCAR-3 cells both in the presence and absence of ES, while it did not cause toxicity in healthy human fibroblasts. In comparison to controls, cancer cells treated with SsnB showed a significant drop in the levels of S1P, PI3K, p-AKT, and p-mTOR. In cancer cells cultured with SsnB, a significant increase in intracellular concentrations of C16-C24 CERs and apoptosis was observed. **Conclusions:** SsnB downregulated the levels of S1P, PI3K, p-AKT, and p-mTOR while reducing cell proliferation and promoting ceramide buildup and apoptosis.

## 1. Introduction

As of 2022, breast cancer remains the most frequently diagnosed cancer among women worldwide, with 2.3 million new cases reported. It accounted for over 670,000 deaths globally in that year. Breast cancer is the most common cancer in women across 157 out of 185 countries, highlighting its global prevalence. Incidence rates are generally higher in countries with high Human Development Index (HDI) scores, where 1 in 12 women are expected to be diagnosed in their lifetime [1]. In 2022, there were approximately 324,603 new cases of ovarian cancer reported globally. This cancer type accounts for an age-standardized incidence rate (ASR) of 6.7 per 100,000 women [1]. Despite advancements in treatment, ovarian cancer remains challenging to manage, particularly in lower-income regions where access to specialized care is limited. Ovarian cancer ranks among the more serious cancers for women, given its high mortality rate, and the number of cases is expected to increase, especially in low- and middle-income countries due to limited access to screening and treatment options.

Recent research highlights that polyphenols, such as curcumin, resveratrol, quercetin, and epigallocatechin gallate (EGCG), are increasingly being explored in combination with chemotherapy for treating breast [2,3] and ovarian cancers [4,5]. These natural compounds offer synergistic benefits by enhancing the efficacy of chemotherapeutic agents like paclitaxel, cisplatin, and 5-fluorouracil [2,3,4,5]. Polyphenols can reduce drug resistance, promote apoptosis, and inhibit cancer cell proliferation. Additionally, they help alleviate the toxic side effects of chemotherapy, improving patient outcomes and quality of life [2,3,4,5]. Research supports that combining these bioactive compounds with standard chemotherapy offers promising results, especially for difficult-to-treat cancers like triple-negative breast cancer and recurrent ovarian cancer [2,3,4,5].

A novel bioactive substance called SsnB was isolated from *Sparganium stoloniferum* and is classified as a polyphenol [6].

**Scheme 1:**
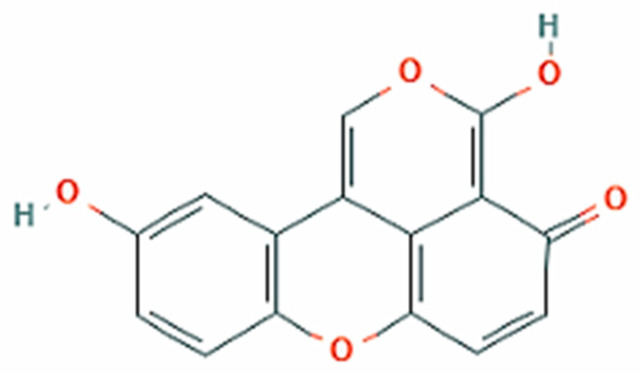
Structure of Sparstolonin B [7].

Its structure, as shown above in Scheme 1 [7] (data are computed by PubChem, https://pubchem.ncbi.nlm.nih.gov, accessed on 13 November 2024), shares similarities with isocoumarins and xanthones, which are known classes of polyphenolic compounds [8]. This chemical composition contributes to its anti-inflammatory and anti-angiogenic properties, which are being explored for cancer treatment and other inflammatory conditions [9]. SsnB acts by inhibiting toll-like receptor signaling, reducing inflammatory cytokine expression, and disrupting cell cycle progression, making it a promising candidate for therapeutic applications [10]. SsnB has been shown to block the PI3K/AKT pathway by inhibiting ROS status in prostate cancer cells [11]. While the effects of SsnB on several cancer types, including neuroblastoma, have been studied [6], specific research on breast or ovarian cancer is limited. Given its mechanism of action—targeting inflammatory pathways and regulating cell survival—there is potential for SsnB to be relevant in breast and ovarian cancer treatments, but further investigations are needed.

Human breast (MCF-7) and ovarian cancer cell lines (OVCAR-3) used in this study are estrogen receptor-positive cells [12]. Estrogen-dependent cell proliferation occurs via the phosphoinositol-3 kinase (PI3K)/serine threonine protein kinase (AKT)/Mechanistic Target of Rapamycin (mTOR) pathway [13]. As a result of the binding of estrogen to its receptor, PI3K is activated and activates AKT. The active AKT phosphorylates a protein called mTOR, which ensures cell proliferation and survival [13]. The PI3K/AKT/mTOR signaling pathway stimulates proliferation and tumor growth in both ovarian and breast cancer cells [14,15].

In hormone-dependent cancers, PI3K/AKT/mTOR signaling is often overactive due to mutations or amplification of pathway components such as PI3K mutations or PTEN loss, resulting in unchecked cell growth and resistance to apoptosis [16]. Furthermore, hormone receptor status, namely ER receptor positivity, is linked to the possible effectiveness of PI3K inhibitors in ER+ breast cancer since PIK3CA mutations are more prevalent in this subtype [17]. More specifically, patients with breast cancer who have lost PTEN function are more likely to benefit therapeutically from PI3K inhibitors [18]. The PI3K/AKT/mTOR signaling pathway also crosstalks with estrogen and androgen receptors, amplifying their transcriptional activity and promoting cancer cell proliferation [19]. Given its centrality to survival and proliferation, the PI3K/AKT/mTOR pathway is a target for cancer therapies. PI3K inhibitors, AKT inhibitors, and mTOR inhibitors (e.g., everolimus) are being tested and used in hormone-dependent cancers [20]. Additionally, combining PI3K/AKT/mTOR inhibitors with hormone therapies (like aromatase inhibitors or anti-androgens) has shown promise, as this dual approach can overcome resistance and lead to better treatment outcomes [21].

SsnB has been shown to block the PI3K/AKT pathway by inhibiting the formation of reactive oxygen metabolites (ROS) [11]. Ceramide is also known to inhibit the PI3K/AKT pathway [22]. Based on this information, we hypothesized that SsnB could suppress estrogen-dependent cell proliferation in breast and ovarian cancer cells and that this effect could develop through the inhibition of the PI3K/AKT/mTOR signaling pathway. The effect of SsnB on ceramide levels in estrogen receptor-positive breast and ovarian cancer cells was also investigated.

## 2. Results

### 2.1. Cell Viability Analysis

#### 2.1.1. Estrogen Proliferation Analysis

**Figure 1:**
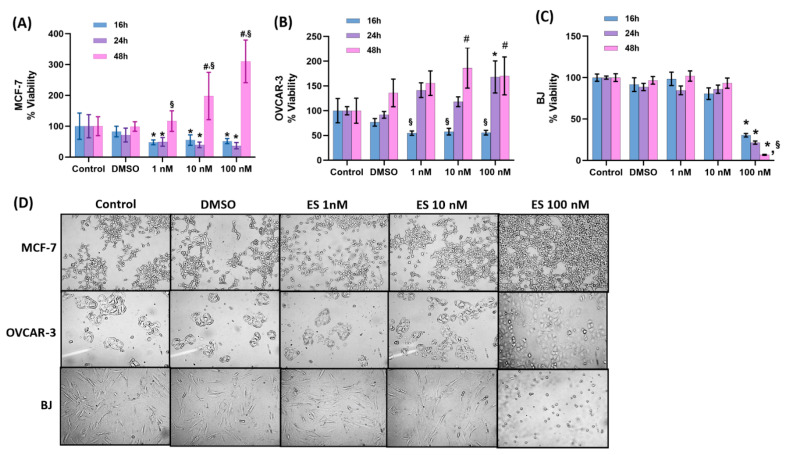
(**A**) Evaluation of cell viability by MTT analysis for 16–48 h in MCF-7 cells. Cells treated with estradiol hemihydrate (ES, 1, 10, and 100 nM) and DMSO, dimethyl sulfoxide (1 μL/mL). Data are representative of 7–8 separate measurements and values are given as mean ± SD. Statistical analysis was performed by two-way ANOVA and differences between groups were determined by Tukey’s multiple comparison test. *, *p* < 0.05, when compared with the control group at the same time periods. #, *p* < 0.05, when compared with all other groups at the same time period. §, *p* < 0.05, when compared with 16 and 24 h within the same dose. (**B**) Evaluation of cell viability by MTT analysis for 16–48 h in OVCAR-3 cells. Data are representative of 6–8 separate measurements and values are given as mean ± SD. Statistical analysis was performed by two-way ANOVA and differences between groups were determined by Tukey’s multiple comparison test. *, *p* < 0.05, when compared with the control and DMSO group at the same time period. #, *p* < 0.05, when compared with the control group at the same time period. §, *p* < 0.05, when compared with 24 and 48 h within the same dose. (**C**) Evaluation of cell viability in BJ cells by MTT analysis for 16–48 h. Data are representative of 6 separate measurements and values are given as mean ± SD. Statistical analysis was performed by two-way ANOVA. Differences between groups were determined by Tukey’s multiple comparison test. *, *p* < 0.05, when compared with all other groups at the same time periods. §, *p* < 0.05, when compared with 16 h within the same dose. (**D**) Light microscope image (10× magnification) of ES-applied MCF-7, OVCAR-3, and BJ cells after 48 h. While no change was observed in the control and DMSO (1 μL/mL) groups, significant proliferation was observed in MCF-7 and OVCAR-3 cells compared to the control as a result of 10 and 100 nM ES applications. It was observed that 100 nM ES application for 48 h caused deterioration in morphology, shrinkage, clustering, and toxicity in BJ cells.

Cell viability analysis following 16, 24, and 48 h of ES (1–100 nM) treatment was performed in MCF-7, OVCAR-3, and non-cancerous BJ fibroblasts (Figure 1). Since the PI3K/AKT/mTOR pathway is typically less active in non-cancerous cells [23], BJ cells provide a contrast to cancer cells, where this pathway is often hyperactive. Cell viability was significantly reduced in MCF-7 cells at 16 and 24 h at all administered ES doses compared to the control groups. In MCF-7 cells, 10 and 100 nM ES at 48 h significantly increased cell proliferation compared to all other groups. All ES doses administered to MCF-7 cells for 48 h significantly increased viability compared to 16 and 24 h applications (Figure 1A).

All ES doses administered to OVCAR-3 cells for 16 h significantly decreased viability compared to 24 and 48 h applications. Treatment of OVCAR-3 cells with 10 and 100 nM ES for 48 h significantly increased cell viability compared to the control group. In OVCAR-3 cells, 100 nM ES treatment for 24 h significantly increased cell proliferation compared to the control and DMSO groups (Figure 1B). In BJ cells, ES 100 nM application at all hours significantly reduced cell proliferation compared to all groups. In addition, 100 nM ES treatment in BJ cells significantly reduced cell viability at 48 h compared to the 16 h-treated group (Figure 1C).

Figure 1D shows light microscope images (10× magnification) of ES applied to MCF-7, OVCAR-3, and BJ cells at 48 h. While no change was observed in the control and DMSO (1 μL/mL) groups, significant proliferation was observed in MCF-7 and OVCAR-3 cells treated with 10 and 100 nM ES compared to the control group. It was observed that the 100 nM ES application for 48 h caused deterioration in morphology, shrinkage, clustering, and toxicity in BJ cells (Figure 1D).

#### 2.1.2. Anti-Proliferative Effects of Sparstolonin B

**Figure 2:**
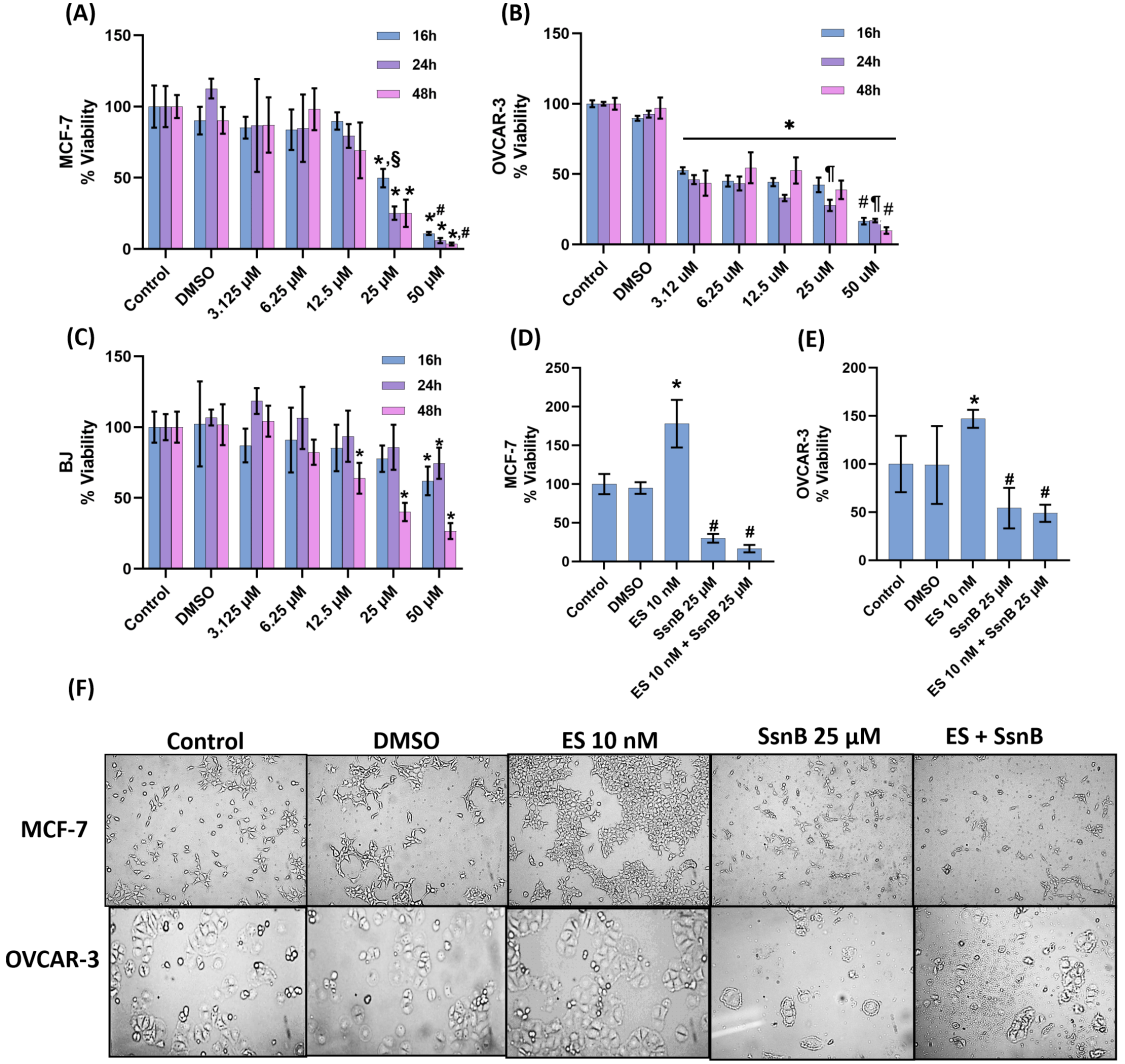
(**A**) The effect of Sparstolonin B(SsnB) on MCF-7 cell viability. Cell viability analysis was performed for 16–48 h. Cells treated with DMSO, dimethyl sulfoxide (1 μL/mL), and cells treated with SsnB. Data are representative of 6–8 separate experiments and values are given as mean ± SD. Statistical analysis was performed by two-way ANOVA and differences between groups were determined by Tukey’s multiple comparison test. *, *p* < 0.05, compared to control, DMSO, 3.125–12.5 µM groups within the same time periods. #, *p* < 0.05, compared to 25 µM group within the same time periods. §, *p* < 0.05 when compared to 24 and 48 h within the same dose. (**B**) The effect of SsnB on OVCAR-3 cell viability. Data are representative of 7 separate experiments and values are given as mean ± SD. Statistical analysis was performed by two-way ANOVA and differences between groups were determined by Tukey’s multiple comparison test. *, *p* < 0.001, vs. DMSO and control in all incubation periods. #, *p* < 0.001, compared to 3.125–25 µM groups within the same time periods. ¶, *p* < 0.05 vs. 3.125 and 6.25 µM groups within the same period. (**C**) Effect of SsnB on BJ cell viability. Data are representative of 7–8 separate experiments and values are given as mean ± SD. Statistical analysis was performed by two-way ANOVA and differences between groups were determined by Tukey’s multiple comparison test. *, *p* < 0.05, vs. all groups in the same time periods. (**D**) Effect of 24 h SsnB treatment on cell viability in MCF-7 cells during 48 h ES proliferation. Data are representative of 8 separate experiments and values are given as mean ± SD. Cells treated with DMSO (1 μL/mL), estradiol hemihydrate (ES, 10 nM), and SsnB (25 μM). Incubation with SsnB started 24 h after ES treatment and was continued for 24 h. Statistical analysis was performed by one-way ANOVA and differences between groups were determined by Holm–Sidak’s multiple comparison test. *, *p* < 0.0001, when compared to all groups. #, *p* < 0.0001, compared with control, DMSO, and ES 10 nM groups. (**E**) Effect of 24 h SsnB treatment on cell viability in OVCAR-3 cells during 48 h ES proliferation. Data are representative of 8 separate experiments and values are given as mean ± SD. Statistical analysis was performed by one-way ANOVA and differences between groups were determined by Holm–Sidak multiple comparison test. *, *p* < 0.05, compared with all groups. #, *p* < 0.05, compared with control, DMSO, and ES 10 nM groups. (**F**) Light microscope image (10× magnification) of MCF-7 and OVCAR-3 cells after 48 h of ES (10 nM) application. Incubation with SsnB started 24 h after ES treatment and was continued for 24 h. Significant proliferation was observed in MCF-7 and OVCAR-3 cells compared to the control as a result of 10 nM ES application. SsnB application significantly decreased cell proliferation and caused significant changes in cell morphology in MCF-7 and OVCAR-3 cells compared to the control and ES groups. ES + SP application was observed to cause deterioration in morphology, shrinkage, clustering, and toxicity in MCF-7 and OVCAR-3 cells.

Cell viability analysis following 16, 24, and 48 h of SsnB (3.125–50 µM) treatment was performed in MCF-7, OVCAR-3, and non-cancerous BJ fibroblasts (Figure 2). SsnB applied at 3.125–12.5 µM doses did not cause a significant reduction in MCF-7 cell viability throughout all time periods. Treatment with 25 and 50 µM SsnB caused a significant decline in cell viability throughout all test periods compared to all other groups. In addition, 25 µM SsnB treatment applied for 24 and 48 h significantly decreased cell viability compared to 16 h treatment within the same dose group. Treatment with 50 µM SsnB for 16 and 48 h significantly reduced cell viability compared to 25 µM within the same time periods (Figure 2A).

In OVCAR-3 cells, all SsnB doses applied throughout all tested time periods significantly reduced cell viability compared to the control and DMSO groups. Moreover, 16 and 48 h SsnB treatment at 50 µM significantly decreased cell viability when compared to 3.125–25 µM doses within the same time periods. In Ovcar-3 cells, 25 and 50 µM SsnB treatment applied for 24 h significantly reduced cell viability compared to 3.125–6.25 µM groups within the same time periods (Figure 2B). In BJ cells, 12.5 µM and 25 µM SsnB treatment applied for 48 h and 50 µM SsnB treatment applied for all time points significantly reduced cell viability compared to all other groups (Figure 2C). In MCF-7 and OVCAR-3 cells, 48 h of 10 nM ES administration significantly increased cell proliferation compared to all other groups. SSnB administration of 25 μM alone significantly reduced viability in both MCF-7 and OVCAR-3 cells compared to the control, DMSO, and ES 10 nM groups. Similarly, 24 h of 25 μM SsnB treatment, which was started following 24 h of 10 nM ES administration, reduced cell viability (Figure 2D,E).

Figure 2F shows the morphological changes in SsnB and ES administration in MCF-7 and OVCAR-3 cell groups. While no change was observed in cells after 24 h of DMSO (1 μL/mL) treatment, significant proliferation was observed in MCF-7 and OVCAR-3 cells compared to the control as a result of the 10 nM ES application. SsnB application significantly decreased cell proliferation and caused significant changes in the cell morphology of MCF-7 and OVCAR-3 cells compared to the control and ES groups. Incubation with SsnB started 24 h after ES treatment and was continued for 24 h. SsnB treatment alone and ES + SsnB applications were observed to cause a deterioration in morphology, a loss of adherence, shrinkage, clustering, and toxicity in MCF-7 and OVCAR-3 cells.

### 2.2. Proliferating Cell Nuclear Antigen Levels

**Figure 3:**
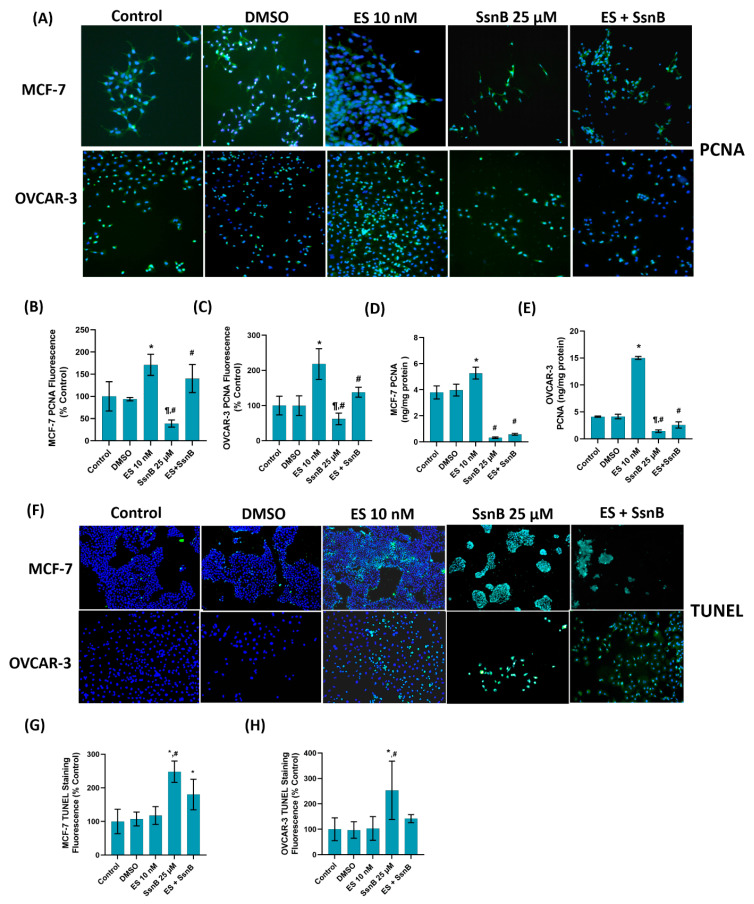
(**A**) Representative immunofluorescent staining of proliferating cell nuclear antigen (PCNA) in MCF-7 and OVCAR-3 cells treated with either DMSO (1 μL/mL), ES (10 nM), or SsnB (25 μM). 10× magnification. Incubation with SsnB started 24 h after ES treatment and was continued for 24 h in the ES + SsnB group. (**B**) Quantitation of PCNA fluorescence staining in MCF-7 cells by ImageJ software (version 1.53k). Data shown are representative of 10 separate measurements and values are given as mean ± SD. Statistical analysis was performed by one-way ANOVA and differences between groups were determined by Tukey’s multiple comparisons analysis. *, *p* < 0.0001, vs. control and DMSO groups. #, *p* < 0.005, vs. control, DMSO, and ES 10 nM groups. ¶, *p* <0.001 vs. ES + SsnB group. (**C**) Quantitation of PCNA fluorescence staining in OVCAR-3 cells by ImageJ software. Data shown are representative of 10 separate measurements and values are given as mean ± SD. Statistical analysis was performed by one-way ANOVA and differences between groups were determined by Tukey’s multiple comparisons analysis. *, *p* < 0.0001, vs. control and DMSO groups. #, *p* < 0.05, vs. control, DMSO, and ES 10 nM groups. ¶, *p* <0.001 vs. ES + SsnB group. (**D**) PCNA protein levels in MCF-7 cells. Data shown are representative of 10 separate measurements and values are given as mean ± SD. Statistical analysis was performed by one-way ANOVA and differences between groups were determined by Tukey’s multiple comparisons analysis. *, *p* < 0.0001, vs. control and DMSO groups. #, *p* < 0.0001, vs. control, DMSO, and ES 10 nM groups. (**E**) PCNA protein levels in OVCAR-3 cells. Data shown are representative of 10 separate measurements and values are given as mean ± SD. Statistical analysis was performed by one-way ANOVA and differences between groups were determined by Tukey’s multiple comparisons analysis. *, *p* < 0.0001, vs. control and DMSO groups. #, *p* < 0.0001, vs. control, DMSO, and ES 10 nM groups. ¶, *p* < 0.05 vs. ES + SsnB group. (**F**) Representative immunofluorescent staining of TUNEL staining in MCF-7 and OVCAR-3 cells. 40× objective lens was used to obtain double-labeled images. (**G**) Quantitation of TUNEL staining in MCF-7 cells with the ImageJ program. Values mean ± SD (*n* = 10). Statistical analysis was performed by one-way ANOVA and differences between groups were determined by Tukey’s multiple comparisons analysis. *, *p* < 0.001, vs. control, DMSO, and ES 10 nM groups. #, *p* < 0.001, vs. ES + SsnB. (**H**) Quantitation of TUNEL staining in OVCAR-3 cells with ImageJ program. Values are mean ± SD (*n* = 10). One-way ANOVA and Tukey’s multiple comparisons were used to determine statistical significance. *, *p* < 0.0001, vs. control, DMSO, and ES 10 nM groups. #, *p* < 0.05, vs. ES + SsnB.

Figure 3A shows PCNA immunofluorescence staining in MCF-7 and OVCAR-3 cells. The quantitation of PCNA fluorescence staining showed that the PCNA protein was significantly increased in MCF-7 (Figure 3B) and OVCAR-3 (Figure 3C) cells treated with 10 nM ES compared to the control, DMSO, and SsnB groups. SsnB (25 µM) treatment alone or SsnB (25 µM) treatment following 24 h ES (10 nM) incubation significantly decreased PCNA immunostaining compared to the 10 nM ES groups. In addition, PCNA immunostaining was significantly reduced in the SsnB treatment group as compared to ES + SsnB. ELISA analysis of PCNA protein levels in MCF-7 (Figure 3D) and OVCAR-3 (Figure 3E) cells confirmed the immunofluorescence staining and showed that the amount of the PCNA protein was significantly increased in cancer cells incubated with 10 nM ES for 48 h compared to all other experimental groups. Treatment with 25 µM SsnB for 24 h significantly decreased cell proliferation compared to all other groups. In addition, PCNA ELISA measurements in OVCAR-3 cells also confirmed significantly reduced levels in the SsnB treatment group as compared to ES + SsnB (Figure 3E).

### 2.3. Apoptotic Cells (TUNEL)

Figure 3F shows TUNEL immunofluorescence staining in MCF-7 and OVCAR-3 cells. There was no discernible difference in the number of apoptotic cells between the control, DMSO, and ES groups. A significant increase in the number of apoptotic cells was observed in the SsnB group compared to all other groups. The combination of ES + SsnB treatment in MCF-7 cells also increased the degree of apoptosis compared to the control, DMSO, and ES 10 nM groups; however, it had no effect on the apoptotic cell number in OVCAR-3 cells (Figure 3G,H).

### 2.4. PI3K, p-AKT, and p-mTOR Levels in MCF-7 and OVCAR-3 Cells

**Figure 4:**
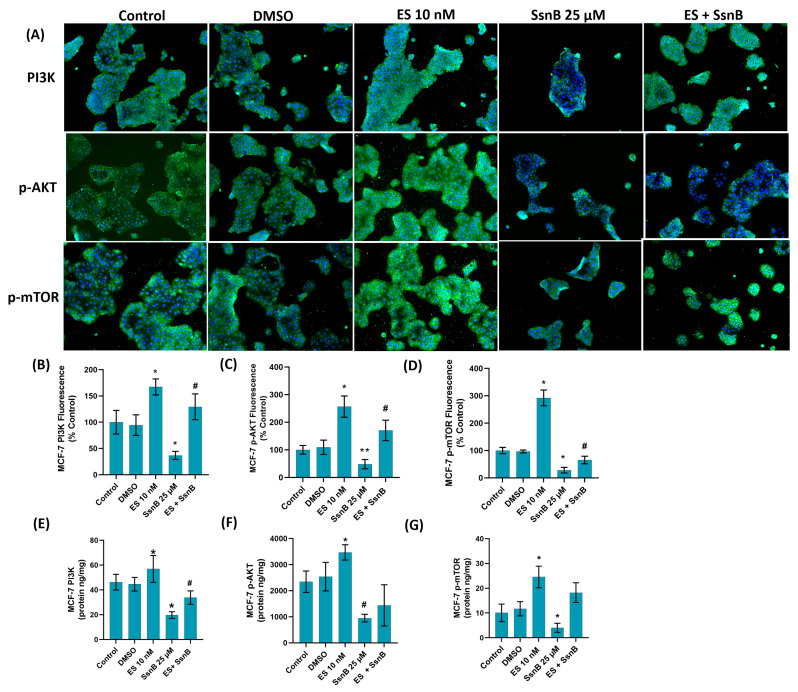
(**A**) Representative immunofluorescent staining of phosphatidylinositol 3-kinase (PI3K), phospho (Ser473) protein kinase AKT (p-AKT), and phospho (Ser2448) mammalian target of rapamycin (p-mTOR) in MCF-7 cells treated with either DMSO (1 μL/mL), ES (10 nM), or SsnB (25 μM). 10× magnification. Incubation with SsnB started 24 h after ES treatment and was continued for 24 h in the ES + SsnB group. (**B**) Quantitation of PI3K fluorescence staining in MCF-7 cells by ImageJ software. Data shown are representative of 9–10 separate measurements and values are given as mean ± SD. Statistical analysis was performed by one-way ANOVA and differences between groups were determined by Tukey’s multiple comparisons analysis. *, *p* < 0.0001, vs. all groups. #, *p* < 0.05, vs. all groups. (**C**) Quantitation of p-AKT fluorescence staining in MCF-7 cells by ImageJ software. Data shown are representative of 9–10 separate measurements and values are given as mean ± SD. Statistical analysis was performed by one-way ANOVA and differences between groups were determined by Tukey’s multiple comparisons analysis. *, *p* < 0.0001, vs. all groups. **, *p*< 0.05 vs. all groups. #, *p* < 0.05, vs. all groups. (**D**) Quantitation of p-mTOR fluorescence staining in MCF-7 cells by ImageJ software. Data shown are representative of 10 separate measurements and values are given as mean ± SD. Statistical analysis was performed by one-way ANOVA and differences between groups were determined by Tukey’s multiple comparisons analysis. *, *p* < 0.0001, vs. all groups. #, *p* < 0.001, vs. all groups. (**E**) PI3K protein levels in MCF-7 cells. Data shown are representative of 7 separate measurements and values are given as mean ± SD. Statistical analysis was performed by one-way ANOVA and differences between groups were determined by Tukey’s multiple comparisons analysis. *, *p* < 0.05, vs. all groups. #, *p* < 0.05, vs. control and DMSO. (**F**) p-AKT protein levels in MCF-7 cells. Data shown are representative of 7 separate measurements and values are given as mean ± SD. Statistical analysis was performed by one-way ANOVA and differences between groups were determined by Tukey’s multiple comparisons analysis. *, *p* < 0.05, vs. all groups. #, *p* <0.001 vs. control, DMSO, and ES 10 nM. (**G**) p-mTOR protein levels in MCF-7 cells. Data shown are representative of 7 separate measurements and values are given as mean ± SD. Statistical analysis was performed by one-way ANOVA and differences between groups were determined by Tukey’s multiple comparisons analysis. *, *p* < 0.05, vs. all groups.

**Figure 5:**
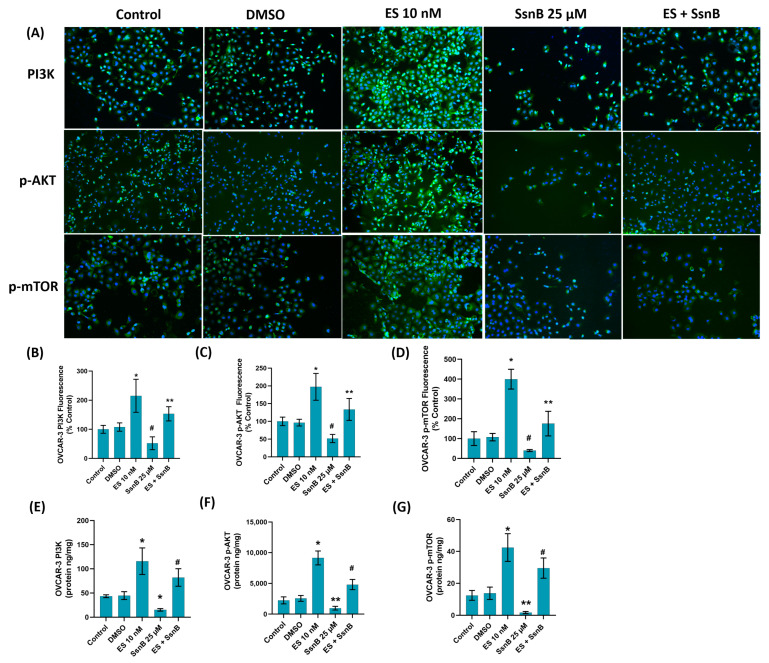
(**A**) Representative immunofluorescent staining of phosphatidylinositol 3-kinase (PI3K), phospho (Ser473) protein kinase AKT (p-AKT), and phospho (Ser2448) mammalian target of rapamycin (p-mTOR) in OVCAR-3 cells treated with either DMSO (1 μL/mL), ES (10 nM), or SsnB (25 μM). 10× magnification. Incubation with SsnB started 24 h after ES treatment and was continued for 24 h in the ES + SsnB group. (**B**) Quantitation of PI3K fluorescence staining in OVCAR-3 cells by ImageJ software. Data shown are representative of 10 separate measurements and values are given as mean ± SD. Statistical analysis was performed by one-way ANOVA and differences between groups were determined by Tukey’s multiple comparisons analysis. *, *p* < 0.001, vs. all groups. **, *p* < 0.05, vs. control and DMSO. #, *p* < 0.05, vs. all groups. (**C**) Quantitation of p-AKT fluorescence staining in OVCAR-3 cells by ImageJ software. Data shown are representative of 10 separate measurements and values are given as mean ± SD. Statistical analysis was performed by one-way ANOVA and differences between groups were determined by Tukey’s multiple comparisons analysis. *, *p* < 0.0001, vs. all groups. **, *p* < 0.05 vs. control and DMSO. #, *p* < 0.001, vs. all groups. (**D**) Quantitation of p-mTOR fluorescence staining in OVCAR-3 cells by ImageJ software. Data shown are representative of 10 separate measurements and values are given as mean ± SD. Statistical analysis was performed by one-way ANOVA and differences between groups were determined by Tukey’s multiple comparisons analysis. *, *p* < 0.0001, vs. all groups. **, *p* < 0.05, vs. control and DMSO #, *p* < 0.05, vs. all groups. (**E**) PI3K protein levels in OVCAR-3 cells. Data shown are representative of 7 separate measurements and values are given as mean ± SD. Statistical analysis was performed by one-way ANOVA and differences between groups were determined by Tukey’s multiple comparisons analysis. *, *p* < 0.05, vs. all groups. #, *p* < 0.05, vs. control and DMSO. (**F**) p-AKT protein levels in OVCAR-3 cells. Data shown are representative of 7 separate measurements and values are given as mean ± SD. Statistical analysis was performed by one-way ANOVA and differences between groups were determined by Tukey’s multiple comparisons analysis. *, *p* < 0.0001, vs. all groups. **, *p* < 0.05 vs. all groups. #, *p* < 0.001 vs. control and DMSO. (**G**) p-mTOR protein levels in OVCAR-3 cells. Data shown are representative of 7 separate measurements and values are given as mean ± SD. Statistical analysis was performed by one-way ANOVA and differences between groups were determined by Tukey’s multiple comparisons analysis. *, *p* < 0.001, vs. all groups. **, *p* < 0.05 vs. all groups. #, *p* < 0.001 vs. control and DMSO.

Figure 4A and Figure 5A show PI3K, p-AKT, and p-mTOR immunofluorescence staining in MCF-7 and OVCAR-3 cells, respectively. The quantitation of PI3K fluorescence staining and PI3K ELISA measurements showed a significant increase in both MCF-7 (Figure 4B and Figure 4E, respectively) and OVCAR-3 (Figure 5B and Figure 5E, respectively) cells in the 10 nM ES groups compared to all other groups. SsnB (25 µM) treatment alone decreased PI3K immunostaining and protein levels in both MCF-7 (Figure 4B and Figure 4E, respectively) and OVCAR-3 (Figure 5B and Figure 5E, respectively) cells compared to all other groups. The increase in PI3K levels following 10 nM ES administration was attenuated following SSnB treatment in the ES + SSnB group, and this decrease was significant in both MCF-7 and OVCAR-3 cells compared to ES 10 nM.

The quantitation of p-AKT fluorescence staining and p-AKT ELISA measurements showed a significant increase in both MCF-7 (Figure 4C and Figure 4F, respectively) and OVCAR-3 (Figure 5C and Figure 5F, respectively) cells in the 10 nM ES groups compared to all other groups. SsnB (25 µM) treatment alone decreased p-AKT immunostaining and protein levels in both MCF-7 (Figure 4C and Figure 4F, respectively) and OVCAR-3 (Figure 5C and Figure 5F, respectively) cells compared to all other groups. The increase in p-AKT levels following 10 nM ES administration was attenuated following SSnB treatment in the ES+ SSnB group, and this decrease was significant in both MCF-7 and OVCAR-3 cells compared to ES 10 nM.

The quantitation of p-mTOR fluorescence staining and p-mTOR ELISA measurements showed a significant increase in both MCF-7 (Figure 4D and Figure 4G, respectively) and OVCAR-3 (Figure 5D and Figure 5G, respectively) cells in the 10 nM ES groups compared to all other groups. SsnB (25 µM) treatment alone decreased p-mTOR immunostaining and protein levels in both MCF-7 (Figure 4D and Figure 4G, respectively) and OVCAR-3 (Figure 5D and Figure 5G, respectively) cells compared to all other groups. The increase in p-mTOR levels following 10 nM ES administration was attenuated following SSnB treatment in the ES + SSnB group, and this decrease was significant in both MCF-7 and OVCAR-3 cells compared to ES 10 nM.

### 2.5. Sphingolipid Levels

Sphingolipid levels measured in MCF-7 and OVCAR-3 cancer cells are shown in Table 1. A significant increase in S1P levels was detected in MCF-7 and OVCAR-3 cells treated with 10 nM ES for 48 h compared to all other groups. Incubation with 25 µM SsnB for 24 h significantly decreased S1P levels in both MCF-7 cells and OVCAR-3 cells compared to all other groups. The increase in S1P levels following 10 nM ES administration was attenuated following SSnB treatment in the ES+ SSnB group, and this decrease was significant in both MCF-7 and OVCAR-3 cells compared to ES 10 nM. Incubation with 25 µM SsnB for 24 h significantly increased C18–C24 ceramide levels in both MCF-7 cells and OVCAR-3 cells compared to all other groups. C18–24 levels were also increased following SSnB treatment in the ES + SSnB group, and this increase was significant in both MCF-7 and OVCAR-3 cells compared to the control, DMSO, and ES 10 nM groups.

## 3. Discussion

The results of this study showed that the treatment of MCF-7 and OVCAR-3 cancer cells with 10 nM estradiol hemihydrate for 48 h significantly increased proliferation. Several studies have reported the proliferative effect of estradiol, specifically at a concentration of 10 nM, on MCF-7 breast cancer cells [24,25]. Estradiol binds to estrogen receptors (primarily ERα in MCF-7 cells), leading to the increased transcription of genes involved in cell proliferation [26]. This concentration of estradiol has been linked to the upregulation of genes that promote cell cycle progression and the downregulation of inhibitory miRNAs like miR-21, which affects tumor suppressor pathways [27]. In one study, treatment with 10 nM estradiol enhanced cell growth and demonstrated receptor-specific effects, highlighting the importance of ERα and ERβ in proliferation and gene regulation [28]. The interaction of estradiol with co-factors like AP-1 transcription factors further underlined its role in cell proliferation through complex regulatory mechanisms [29]. These findings confirm that estradiol at physiologically relevant concentrations plays a significant role in promoting the growth of hormone-responsive breast cancer cells like MCF-7, reinforcing its importance in breast cancer research and therapeutic targeting strategies.

Studies also suggest that estradiol influences proliferation in ovarian cancer cell lines like OVCAR-3. Research shows that estradiol, in the nanomolar range, affects various aspects of cancer cell behavior, including growth and receptor expression [30]. In experiments with OVCAR-3 cells, researchers often use estradiol to investigate estrogen receptor-mediated mechanisms. These studies sometimes compare effects across multiple concentrations, such as 1 nM, 10 nM, and 100 nM, reflecting dose-dependent responses related to estrogen sensitivity and signaling pathways [31].

The incubation of MCF-7 and OVCAR-3 cells with 25 µM SsnB for 24 h significantly reduced cell proliferation both in the presence and absence of ES. Studies have shown that SsnB can significantly reduce the proliferation of cancer and endothelial cells [32]. SsnB has been reported to inhibit cancer-related processes such as cell migration, invasion, and angiogenesis by interfering with the cell cycle, particularly by halting cells at the G1 or G2/M checkpoints [32]. SsnB concentrations ranging from 10 µM to 100 µM have shown strong antiproliferative effects in various cell types, including neuroblastoma [6] and prostate cancer models [11]. To the best of our knowledge, this study shows for the first time that SSnB inhibits estrogen-dependent proliferation in MCF-7 and OVCAR-3 cancer cells. This compound also disrupts vascular endothelial cell function, which could be critical for tumors that rely on angiogenesis for growth and metastasis [32].

MCF-7 and OVCAR-3 cells treated with cytotoxic concentrations of SsnB for 24 h showed a significant increase in intracellular levels of C18–C24 ceramides in comparison to controls. This work is the first to assess how SsnB affects the amounts of endogenous sphingolipids in breast and ovarian cancer cells. It appears that current research has not directly linked SsnB to increased ceramide levels. Most studies on Sparstolonin B focus on its anti-inflammatory, anti-proliferative, and anti-angiogenic effects, primarily through modulating Toll-like receptor (TLR) pathways [10] and suppressing NF-κB and STAT1 signaling [33]. The impact of SsnB on lipid metabolism or ceramide levels specifically has not been well documented in the studies reviewed. Sparstolonin B is a selective TLR antagonist with potent anti-inflammatory properties [10]. In mouse macrophages stimulated by a TLR4, TLR1/TLR2, or a TLR2/TLR6 ligand, SsnB substantially suppressed inflammatory cytokine expression [10]. Toll-like receptor 4 promotes the survival and invasiveness of breast cancer cells and ovarian cancer cells through the induction of cell proliferation and apoptosis resistance [34,35].

The relationship between TLR signaling and ceramide synthesis in cancer cells is complex. Some research indicates potential crosstalk between TLR signaling and sphingolipid metabolism, including ceramide production, which plays a role in cancer therapy [36]. However, direct examples of TLR antagonists increasing ceramide synthesis are not well-established. TLRs control the inflammatory responses in cancer cells, and increased TLR expression has been associated with oncogenesis and the advancement of cancer in cancer cell lines [37]. The relationship between TLR and cancer is still debatable, though. TLRs have the apparent ability to slow the advancement of cancer [38,39], but they have also been shown to accelerate it [40]. These compounds are appealing as therapeutic targets because of the increased expression of TLRs within a tumor. The TLR2/TLR4 agonist (Bacillus Calmette and Guérin) is the most effective TLR ligand in cancer therapy; it has been used to treat bladder cancer for more than thirty years [41]. Proinflammatory chemokines and immunosuppressive cytokines are produced irregularly and uninhibitedly by TLR-4 during tumor progression; this suggests that finding TLR-4 antagonists could be a great way to treat cancer. TLR-4 antagonists, however, carry the potential to jeopardize host immunity. Therefore, the question of whether to target TLR-4 agonists or antagonists for the treatment of cancer remains unresolved in science. To completely understand the roles of TLR-4 agonists and antagonists in different malignancies, more research must be conducted [42]. Our findings suggest that manipulating TLR signaling holds therapeutic promise, with the potential to exploit ceramide’s role in inducing cancer cell death and overcoming drug resistance mechanisms. SsnB’s modulation of the TLR pathway is hypothesized to influence ceramide synthesis, leading to enhanced ceramide-driven apoptosis. This mechanism leverages TLR inhibition to create a pro-apoptotic environment through ceramide accumulation, which, when combined with weakened survival signaling, may selectively induce apoptosis in cancer cells. This dual modulation underscores the potential of SsnB as a targeted therapeutic agent in cancers with activated TLR pathways and dysregulated survival mechanisms.

We found that giving cancer cells a 24 h therapy with 25 µM SsnB significantly lowered their S1P levels. To the best of our knowledge, our work is the first to document decreased S1P levels in SsnB-treated cancer cells. Ceramide and S1P are examples of functional sphingolipid metabolites that play critical roles in the operation of multiple biological pathways that are essential to the pathophysiology of cancer. The current understanding of biologically active sphingolipid production acknowledges their critical roles in the progression and development of cancer. Ceramide is an essential part of the metabolism of sphingolipids and functions as a tumor-suppressive chemical in many malignant cells, inducing anti-proliferative and apoptotic responses [43]. Conversely, S1P triggers processes that turn it into a lipid that promotes cancer [44].

We have seen that PI3K, p-AKT, and p-mTOR proteins were significantly suppressed in cancer cells treated with SsnB compared to the control groups. Our results support previous studies that have shown that SsnB blocks the PI3K/AKT pathway in prostate cancer cells [11]. The PI3K/AKT/mTOR pathway is a critical intracellular signaling pathway that regulates cell growth, survival, metabolism, and proliferation [45]. It is often hyperactivated in cancers, including breast [46] and ovarian cancers [47], contributing to tumor progression, metastasis, and therapy resistance. External signals like insulin, growth factors (e.g., EGF, VEGF), or hormones bind to receptor tyrosine kinases (RTKs) such as HER2 or the insulin receptor. This activates PI3K, a lipid kinase that converts PIP2 to PIP3 on the plasma membrane [48]. PIP3 recruits AKT to the membrane, where it is phosphorylated and activated. Phosphorylated AKT promotes cell survival by inhibiting pro-apoptotic factors and stimulating anti-apoptotic proteins. AKT indirectly activates mTOR, a major regulator of cell growth [48]. In breast cancer, PI3K/AKT/mTOR is frequently activated due to mutations in genes like PIK3CA and the amplification of receptor tyrosine kinases like HER2 [46]. These alterations drive tumor growth and metastasis. Up to 40% of breast cancers with mutations in PIK3CA are estrogen receptor (ER)-positive, contributing to resistance against hormonal therapies. As a result, PI3K/AKT/mTOR inhibitors are being tested to improve treatment outcomes for breast cancer patients, especially those with endocrine resistance [48]. In ovarian cancer, this pathway is similarly implicated in disease progression, chemoresistance, and the epithelial-to-mesenchymal transition (EMT), which allows cancer cells to become more invasive. Targeting PI3K/AKT/mTOR can reduce stem cell-like properties in ovarian cancer cells, which are often associated with relapses and resistance to standard treatments [47]. Drugs like BEZ235, an mTOR/PI3K dual inhibitor, are being investigated to enhance chemotherapy responses by reversing EMT and decreasing markers associated with cancer stem cells [49].

As was previously mentioned, ceramide production stimulation and apoptosis induction are key mechanisms by which SsnB exerts its anticancer effects. Protein kinase C (PKC) is dephosphorylated and inactivated because of ceramide stimulation of protein phosphatase 2A (PP2A) [50]. PKC is a physiological Bcl2 kinase. Bcl2 must be phosphorylated at serine 70 to fully and effectively inhibit apoptosis. Its normal protein phosphatase, PP2A, dephosphorylates Bcl2. When Bcl2 is dephosphorylated by ceramide through PP2A, its anti-apoptotic properties are rendered inactive [50]. A substantial increase in apoptosis was observed in cancer cells treated with 25 µM SsnB for a full day, as revealed by TUNEL analysis. Our results corroborate earlier research suggesting that SsnB elevated apoptosis in pancreatic cancer [51] and in prostate cancer cells [11].

Ceramide is known for its role in promoting apoptosis and inhibiting cellular proliferation. The research hypothesis presented herein suggests that SsnB may increase cellular ceramide levels, either by stimulating ceramide synthesis or inhibiting its degradation. Elevated ceramide levels can trigger apoptosis and may initiate cellular stress responses that oppose cancer cell growth. Ceramide is known to activate the protein PP2A [52], which can downregulate survival pathways, including PI3K/AKT/mTOR [53]. The hypothesis posits that SsnB may also inhibit this pathway directly, which would decrease survival signals within cancer cells, making them more susceptible to cell death. Thus, the hypothesis suggests a synergistic action in which SsnB increases ceramide levels, which either directly or indirectly inhibits the PI3K/AKT/mTOR pathway, leading to enhanced apoptosis and reduced cancer cell viability. This dual mechanism targets both a lipid-signaling molecule, ceramide, and a key survival pathway, PI3K/AKT/mTOR, amplifying the potential anti-cancer effects of SsnB.

In conclusion, SsnB demonstrates significant anticancer potential by reducing cancer cell viability, especially in estrogen-dependent cancers. This effect is achieved through multiple molecular mechanisms: SsnB downregulates key proliferative proteins, including proliferating cell nuclear antigen (PCNA), sphingosine-1-phosphate (S1P), phosphorylated AKT (p-AKT), and phosphorylated mTOR (p-mTOR). These proteins are central to the PI3K/AKT/mTOR signaling pathway, a pathway frequently upregulated in cancer and involved in promoting cell growth, survival, and metabolism. Additionally, SsnB elevates ceramide levels, a bioactive lipid known for its pro-apoptotic properties, which enhances cell death in cancer cells and shifts the cellular environment toward apoptosis over proliferation.

Future research on SsnB should focus on its role in modulating the balance between ceramide and S1P, given that these metabolites are key regulators of cell fate in cancer. Investigating SsnB’s effects on other cell signaling pathways, particularly those associated with hormone receptors in estrogen-dependent cancers, may provide a more comprehensive understanding of its therapeutic potential. Furthermore, the development of SsnB analogs or derivatives could enhance bioavailability and specificity, making it a promising candidate for combination therapies targeting PI3K/AKT/mTOR-driven cancers.

## 4. Materials and Methods

### 4.1. Cell Culture

Estrogen-positive non-metastatic human breast cancer (MCF-7) and non-cancer human fibroblast (BJ) cell lines were obtained from ATCC (Manassas, VA, USA) while human ovarian epithelial cancer (OVCAR-3) cell lines were purchased from İ-Cell (Fengxian Dist, Shanghai, China). MCF-7 cells were grown in Dulbecco’s Modified Eagle’s Medium (DMEM)–F12 (Biowest, Nuaillé, France) while OVCAR-3 and BJ cells were maintained in RPMI-1640 medium (Capricorn; Cat.#RPMI-A, Ebsdorfergrund, Germany) containing 10% fetal bovine serum (FBS) (Gibco, Life Technologies, Grand Island, NY, USA), 1% penicillin and streptomycin (Gibco), and antifungal amphotericin B (1%, Gibco) and supplemented with 5% sodium bicarbonate. All cell cultures were kept in an incubator set to 37 °C with 5% carbon dioxide and 95% humidity. Once the cells reached 70–80% confluency, they were transferred to new flasks after being detached from the flask surface using trypsin (0.05%)-EDTA (0.02%) solution (Gibco).

### 4.2. Estradiol and Sparstolonin B Treatment

Cell proliferation was stimulated using estradiol hemihydrate (ESTROFEM, MW: 562.8 g/mol, Novo Nordisk Pharmaceutical Industry, Istanbul, Turkey). A stock concentration of 3.6 mM of estrogen hemihydrate was prepared by dissolving a 2 mg tablet in 1 mL of DMSO. This stock was further diluted with the cell culture medium to create a 1 mM intermediate stock solution. The final estrogen concentrations tested in the MTT assay were 1, 10, and 100 nM. The MTT assay results helped define the optimal dose and period of estrogen treatment for MCF-7, OVCAR-3, and BJ cells. Sparstolonin B (purity ≥ 99.0% and MW: 268.32 g/mol) was purchased from Sigma-Aldrich (St. Louis, MO, USA). To prepare a 10 mM stock solution, 5 mg of Sparstolonin B was dissolved in 1864 µL of DMSO. This stock was then diluted with a cell culture medium to form a 1 mM intermediate solution. The final concentrations of Sparstolonin B used in the MTT assay ranged from 3.125 to 50 µM. The results from the MTT assay were used to establish the final dose and period of Sparstolonin B treatment in MCF-7, OVCAR-3, and BJ cells.

### 4.3. Cell Viability

An MTT assay (3-(4,5-dimethylthiazol-2-yl)-2,5-diphenyltetrazolium bromide) was performed to analyze cell viability. MTT (Gold Biotechnology Inc., St. Louis, MO, USA) was dissolved in a PBS solution (5 mg/mL) and filtered via a 0.22 µm pore size filter. MCF-7, OVCAR-3, and BJ cells were seeded in sterile 96-well plates at a density of 5 × 10^3^ cells per 100 µL of medium. Cells were cultured overnight to allow attachment before being treated with the cell culture medium containing either 1 µL/mL DMSO, 1–10–100 nM ES, or 3.125–50 µM SsnB. After incubation periods of 16, 24, and 48 h, the MTT protocol was initiated. The incubation medium was removed, and 90 µL of the fresh medium along with 10 µL of MTT were added to each well. Following 2 h incubation at 37 °C, the purple formazan crystals that had formed were dissolved by the addition of 100 µL of DMSO. Absorbance was measured at 570 and 690 nm using a MicroQuant plate reader (Bio-Tek Instruments Inc., Charlotte, VT, USA). The absorbance from the control group was considered as 100% cell viability, and cell viability (%) was calculated using the formula: Cell viability (%) = (Abs sample/Abs control) × 100.

Five different experimental groups were created according to the MTT cell viability results: (1) Control (only medium); (2) DMSO (cells incubated with 1 µL/mL DMSO for 24 h); (3) ES (cells treated with 10 nM ES for 48 h); (4) SsnB (cells incubated with 25 µM SsnB for 24 h); and (5) ES + SsnB (incubation with 25 µM SsnB started 24 h after 10 nM ES treatment and was continued for 24 h).

### 4.4. Immunostaining

Approximately 100,000 cells per well (MCF-7 or OVCAR-3) were plated on 8-well chamber slides (Merck Millipore, Cork, Ireland). For cell adhesion, the chamber slides were incubated for the entire night at 37 °C and 5% CO_2_. After the cells achieved 70% confluency, the culture medium was switched out for the treatment medium, and the incubation process lasted for 24 h. After the incubation period, the medium was removed, and cells underwent two washes using 0.01 M cold PBS. A freshly made 250 μL solution of 4% paraformaldehyde (Sigma-Aldrich, St. Louis, MO, USA) was added, and cells were fixed for ten minutes at room temperature. After aspirating the fixing solution, the cells underwent two PBS washes. To initiate the permeabilization process, 300 μL of PBS containing 0.2% Triton X-100 (Sigma-Aldrich, St. Louis, MO, USA) was added and incubated at room temperature for 30 min. The cells were then washed five times with cold PBS and blocked with 5% normal goat serum (NGS from Vector Laboratories, Burlingame, CA, USA). After removing the blocking solution, the cells were treated overnight at 4 °C with primary antibodies against PI3K (#BT-AP07136, BT Bioassay Technology Laboratories, Jiaxing, Zhejiang, China), phospho-AKT (#649001, BioLegend, San Diego, CA, USA), phospho-mTOR (BT-AP07085, BT Bioassay Technology Laboratories, Jiaxing, Zhejiang, China), and PCNA (Cat.#bs-0754R, Bioss Antibodies Inc., Woburn, MA, USA). Each antibody was diluted 1:200 in 200 µL of PBS. After 24 h, the wells were washed five times with PBS at room temperature and incubated with 200 µL of Alexa Fluor-488-conjugated goat anti-rabbit secondary antibody (Cat. #ab150077 Abcam, Cambridge, UK) (1:1000 dilution) for 45 min in a dark room. Cells were washed in PBS 3 times for 5 min, and a drop of DAPI (Vector Laboratories Inc., Burlingame, CA, USA) was added for nuclear staining. Finally, the slides were covered with a coverslip without air bubbles, and photographs were taken under a microscope. Slides were viewed using a fluorescence microscope (Olympus IX81, Tokyo, Japan) at ×10 magnification. Alexa Fluor dye was imaged at 488 nm excitation and 505–525 nm emission, while DAPI was imaged at 350 nm excitation and 440–460 nm emission. The fluorescence intensity in MCF-7 and OVCAR-3 cells was analyzed using NIH ImageJ 1.53k software. The corrected total cell fluorescence (CTCF) for individual cells in each group was calculated using the following formula: CTCF = Integrated Density − (Area of selected cell × Background mean fluorescence).

### 4.5. ELISA Measurements

To measure the levels of PCNA, a non-competitive sandwich ELISA kit (Cat. #ELK5141, ELK Biotechnology, Denver, CO, USA) was used. Following treatment regimens for MCF-7 and OVCAR-3 cells, 10^7^ cells/mL were suspended in PBS and underwent ultrasonication. Cell lysates were centrifuged at 1500× *g* for 10 min at 2–8 °C. The PCNA levels in the supernatants were evaluated in compliance with the kit’s instructions. Using a standard curve, the PCNA intensity in the samples was determined at 450 nm and expressed as ng/mg of protein.

Levels of PI3K, phospho-Akt (ser473), and phospho-mTOR were assessed by sandwich ELISA kits (cat. #E0896Hu, cat. #E4531Hu, cat. #E4485Hu, respectively. BT Lab, Bioassay Technology Laboratory; Shanghai, China). MCF-7 and OVCAR-3 cells were treated according to the study groups, and the collected cells (10^7^ cells/mL) were suspended in PBS and then subjected to ultrasonication and centrifugation at 1500× *g* for 10 min at 2–8 °C. Measurements of PI3K, phospho-Akt, and phospho-mTOR were conducted in the supernatants in accordance with the manufacturer’s instructions. The amount of PI3K, phospho-AKT (ser473), and phospho-mTOR in the samples was determined at 450 nm using a standard curve and reported as ng/mg of protein.

### 4.6. Protein Levels

Protein levels were determined at 595 nm via a modified Bradford assay and the Pierce Bradford Plus Protein Assay Reagent (Thermo Fisher Scientific Cat. # 23238, Thermo Fisher, Waltham, MA, USA). Bovine serum albumin was used as the standard.

### 4.7. Apoptotic Cell Analysis

Apoptotic cells were determined using the One-Step TUNEL Test Kit (Elabscience, Cat#E-CK-A320, Houston, TX, USA). A catalytic process by terminal deoxynucleotidyl transferase (TdT) can add fluorescein-labeled dUTP to the exposed 3′-OH ends of damaged DNA. Every reagent and sample was prepared following the instructions included in the test kit. Approximately 100,000 cells per well (MCF-7 or OVCAR-3) were plated on 8-well chamber slides (Merck Millipore, Cork, Ireland). After the cells achieved 70% confluency, the culture medium was switched out for the treatment medium, and the incubation process lasted for 24 h. Following the incubation period, the media was removed, the cells were washed with PBS, and paraformaldehyde fixation was carried out for 20 min at room temperature. DNase I was used to break off DNA in the positive control group, exposing 3′-OH ends. After applying 100 μL of the DNase I solution (200 U/mL), the cells were incubated for 30 min at 37 °C. PBS was used to wash the slides (three times, for 5 min each). Cells used as negative controls were incubated for five minutes at room temperature in the buffer, and then they were washed three times for 5 min each in PBS. Following the completion of the penetration step, 100 μL of the TdT Balancing Buffer was added, and the slides were incubated for 25 min at 37 °C. Upon the conclusion of the incubation period, 50 μL of the Labeling Working Solution was introduced into every slide, followed by 60 min humidified incubation at 37 °C. The TdT enzyme was absent in the working solution used for labeling negative controls. The slides were washed three times with PBS for five minutes each. After separating the chambers and wiping them with a napkin, DAPI was dropped onto the slides, and a spotless coverslip was sealed without any air bubbles. A fully automated Olympus BX61 microscope was used to visualize fluorescence intensity, and ImageJ software (version 1.53k; NIH) was used for analysis.

### 4.8. Sphingolipid Measurements

Two microliters of the 5 µg/mL IS stock solution were added to one milliliter of OVCAR-3 and MCF-7 cell lysates (10 mg protein/mL). Following the vortexing of tubes, 100 μL of distilled water was added, and samples were sonicated for 30 s before being vortexed for 5 min with a 1:1, *v*/*v* ratio of chloroform to methanol. After 30 min of incubation at room temperature, the resultant mixture was centrifuged for 5 min at 2000× *g* to extract the supernatants. After adding 125 μL of chloroform and 125 μL of distilled water to the supernatants, they were vortexed and allowed to remain at room temperature for half an hour. Following the incubation period, roughly 500 µL of the uppermost organic layer was moved to a fresh glass tube, and the volatilization procedure was executed with a continuous nitrogen supply (VLM, Bielefeld, Germany). Following the dissolution of the dried residues in 100 μL of methanol–formic acid (99.9:0.1), the samples were moved to insert vials and prepared for LC-MS/MS analysis. The procedures previously outlined for LC-MS/MS measurements were followed [54].

### 4.9. Statistical Analysis

The statistical programs SigmaPlot for Windows and GraphPad Prism 9.00 (Systat Software, Inc., Chicago, IL, USA) were used to analyze the data. *p* values less than 0.05 were deemed statistically significant. The statistical analysis for each measurement is explained in depth in the figure and table legends. Before comparing the groups using statistical analysis, a normality test was run. We used a nonparametric test when the data were not normally distributed. With its ability to control Type I errors across multiple comparisons, provide thorough pairwise comparisons following ANOVA, assume equal variances, and treat all comparisons equally, Tukey’s test is a robust option for assessing the differences between cell viability levels across various treatment conditions in a balanced and statistically rigorous manner, making it appropriate for analyzing cell viability data in studies with multiple treatment groups [55].

## Figures and Tables

**Table 1:** Sphingolipid levels in MCF-7 and OVCAR-3 cells.

Sphingolipids	Control	DMSO	ES 10 nM	SsnB 25 µM	ES + SsnB
16:0 SM (d18:1/16:0)					
MCF-7	124.99 ± 5.22	156.82 ± 27.99	165.75 ± 25.34	153.98 ± 13.82	143.83 ± 30.33
OVCAR-3	157.40 ± 8.14	170.01 ± 13.15	143.50 ± 9.06	133.11 ± 28.17	158.64 ± 26.93
18:0 SM (d18:1/18:0)					
MCF-7	67.44 ± 13.12	60.60 ± 7.90	65.42 ± 24.01	61.71 ± 13.20	66.73 ± 7.84
OVCAR-3	65.09 ± 14.51	59.68 ± 2.59	66.93 ± 17.35	57.40 ± 6.20	69.15 ± 22.34
24:0 SM (d18:1/24:0)					
MCF-7	45.42 ± 5.91	38.30 ± 3.54	41.46 ± 5.51	39.846 ± 6.82	36.15 ± 4.46
OVCAR-3	44.08 ± 4.77	41.5 ± 2.005	41.16 ± 3.11	47.386 ± 6.37	44.83 ± 8.98
C16 Ceramide (d18:1/16:0)					
MCF7	71.37 ± 9.38	73.71 ± 3.38	71.71 ± 2.11	7.02 ± 3.88	67.21 ± 7.79
OVCAR-3	66.41 ± 7.28	69.63 ± 7.90	69.85 ± 6.76	69.87± 2.26	63.51 ± 14.33
C18 Ceramide (d18:1/18:0)					
MCF-7	11.02 ± 0.77	10.26 ± 0.54	12.33 ± 0.12	27.36 ± 1.13 **	22.73 ± 2.01 *
OVCAR-3	4.59 ± 1.08	6.11 ± 2.16	4.8 ± 0.327	11.77 ± 0.67 *	11.90 ± 2.51 *
C20 Ceramide (d18:1/20:0)					
MCF-7	12.51 ± 1.72	14.77 ± 1.42	15.61 ± 1.76	31.68 ± 0.57 *	30.54 ± 0.79 *
OVCAR-3	4.08 ± 0.36	4.61 ± 0.69	5.16 ± 0.73	33.91 ± 2.53 **	27.73 ± 1.52 *
C22 Ceramide (d18:1/22:0)					
MCF-7	29.83 ± 2.032	27.14 ± 3.11	24.69 ± 1.70	65.98 ± 3.64 *	54.21 ± 2.76 *
OVCAR-3	17.24 ± 0.98	17.44 ± 1.38	17.85 ± 3.77	50.99 ± 0.55 *	48.51 ± 1.76 *
C24 Ceramide (d18:1/24:0)					
MCF-7	39.33 ± 1.09	36.41 ± 2.06	39.76 ± 2.75	98.55 ± 7.38 *	88.77 ± 2.56 *
OVCAR-3	39.56 ± 3.38	37.57 ± 4.61	32.80 ± 5.11	97.56 ± 3.30 *	92.05 ± 4.72 *
S1P					
MCF-7	6.55 ± 0.47	5.87 ± 0.21	16.81 ± 0.29 ^≠^	1.59 ± 0.10 ^$^	8.91 ± 0.14 ^≠≠^
OVCAR-3	14.86 ± 0.22	14.89 ± 0.20	30.23 ± 0.44 ^≠^	6.48 ± 0.22 ^$^	11.43 ± 0.78 ^≠≠^

All values mean ± SD. *n* = 6 for each group. Levels are given as ng/mg protein. SM, sphingomyelin; S1P, sphingosine-1-phosphate; DMSO, Dimethyl Sulfoxide; ES, Estradiol Hemihidrat. Statistical analysis was conducted using One-Way Analysis of Variance and all pairwise multiple comparison procedures were performed using Tukey’s test. *, *p* < 0.01 vs. control, DMSO, ES 10 nM groups. **, *p* < 0.01, vs. control, DMSO, ES 10 nM, and ES + SsnB groups. ≠, *p* < 0.001, vs. control, DMSO, SsnB 25 µM and ES + SsnB groups. ≠≠, *p* < 0.001, vs. control, DMSO, ES 10 nM vs. SsnB 25 µM groups. $, *p* < 0.001, vs. control, DMSO, ES 10 nM, and ES + SsnB groups.

## Data Availability

Data obtained and analyzed in this work are available from the corresponding author upon reasonable request.

## Abbreviations

ASR: age-standardized incidence rate; BJ, human fibroblast; CERs, ceramides; CTCF, corrected total cell fluorescence; DMEM, Dulbecco’s modified eagle’s medium; EGCG, epigallocatechin gallate; ELISA, enzyme-linked immunosorbent assay; ES, estradiol hemihydrate; FBS, fetal bovine serum; MCF-7, human breast cancer; OVCAR-3, ovarian epithelial cancer; PCNA, proliferating cell nuclear antigen, PI3K, phosphoinositol-3 kinase; p-mTOR, phosphorylated mechanistic target of rapamycin; ROS, reactive oxygen metabolites; RTKs, receptor tyrosine kinases; S1P, sphingosine-1-phosphate; SMs, sphingomyelins; SsnB, Sparstolonin B; TLR, Toll-like receptor.

## References

[1] Bray F., Laversanne M., Sung H., Ferlay J., Siegel R.L., Soerjomataram I., Jemal A. (2024). Global cancer statistics 2022: GLOBOCAN estimates of incidence and mortality worldwide for 36 cancers in 185 countries. CA Cancer J. Clin..

[2] Brala C.J., Marković A.K., Kugić A., Torić J., Barbarić M. (2023). Combination Chemotherapy with Selected Polyphenols in Preclinical and Clinical Studies—An Update Overview. Molecules.

[3] Mecca M., Sichetti M., Giuseffi M., Giglio E., Sabato C., Sanseverino F., Marino G. (2024). Synergic Role of Dietary Bioactive Compounds in Breast Cancer Chemoprevention and Combination Therapies. Nutrients.

[4] Sharifi-Rad J., Seidel V., Izabela M., Monserrat-Mequida M., Sureda A., Ormazabal V., Zuniga F.A., Mangalpady S.S., Pezzani R., Ydyrys A. (2023). Phenolic compounds as Nrf2 inhibitors: Potential applications in cancer therapy. Cell Commun. Signal..

[5] Peng Z., Li H., Gao Y., Sun L., Jiang J., Xia B., Huang Y., Zhang Y., Xia Y., Zhang Y. (2024). Sintilimab combined with bevacizumab in relapsed or persistent ovarian clear cell carcinoma (INOVA): A multicentre, single-arm, phase 2 trial. Lancet Oncol..

[6] Kumar A., Fan D., DiPette D.J., Singh U.S. (2014). Sparstolonin B, a Novel Plant Derived Compound, Arrests Cell Cycle and Induces Apoptosis in N-Myc Amplified and N-Myc Nonamplified Neuroblastoma Cells. PLoS ONE.

[7] PubChem. https://pubchem.ncbi.nlm.nih.gov.

[8] Yepuri N., Dhawan R., Cooney M., Pruekprasert N., Meng Q., Cooney R.N. (2019). Sparstolonin B: A Unique Anti-Inflammatory Agent. Shock.

[9] Tuorkey M.J. (2015). Cancer Therapy with Phytochemicals: Present and Future Perspectives. Biomed. Environ. Sci..

[10] Liang Q., Wu Q., Jiang J., Duan J., Wang C., Smith M.D., Lu H., Wang Q., Nagarkatti P., Fan D. (2011). Characterization of Sparstolonin B, a Chinese Herb-derived Compound, as a Selective Toll-like Receptor Antagonist with Potent Anti-inflammatory Properties. J. Biol. Chem..

[11] Liu S., Hu J., Shi C., Sun L., Yan W., Song Y. (2021). Sparstolonin B exerts beneficial effects on prostate cancer by acting on the reactive oxygen species-mediated PI3K/AKT pathway. J. Cell. Mol. Med..

[12] Karpeta A., Gregoraszczuk E. (2017). Differences in the mechanisms of action of BDE-47 and its metabolites on OVCAR-3 and MCF-7 cell apoptosis. J. Appl. Toxicol..

[13] Zhang H.-P., Jiang R.-Y., Zhu J.-Y., Sun K.-N., Huang Y., Zhou H.-H., Zheng Y.-B., Wang X.-J. (2024). PI3K/AKT/mTOR signaling pathway: An important driver and therapeutic target in triple-negative breast cancer. Breast Cancer.

[14] Aziz A.U.R., Farid S., Qin K., Wang H., Liu B. (2018). PIM Kinases and Their Relevance to the PI3K/AKT/mTOR Pathway in the Regulation of Ovarian Cancer. Biomolecules.

[15] Raphael J., Desautels D., Pritchard K.I., Petkova E., Shah P.S. (2018). Phosphoinositide 3-kinase inhibitors in advanced breast cancer: A systematic review and meta-analysis. Eur. J. Cancer.

[16] Tufail M., Hu J.-J., Liang J., He C.-Y., Wan W.-D., Huang Y.-Q., Jiang C.-H., Wu H., Li N. (2024). Predictive, preventive, and personalized medicine in breast cancer: Targeting the PI3K pathway. J. Transl. Med..

[17] Shariati M., Evans K.W., Zheng X., Bristow C.A., Ng P.K.-S., Rizvi Y.Q., Tapia C., Yang F., Carugo A., Heffernan T.P. (2021). Combined inhibition of DDR1 and CDK4/6 induces synergistic effects in ER-positive, HER2-negative breast cancer with PIK3CA/AKT1 mutations. Oncogene.

[18] Dillon L.M., Miller T.W. (2014). Therapeutic Targeting of Cancers with Loss of PTEN Function. Curr. Drug Targets.

[19] Peng Y., Wang Y., Zhou C., Mei W., Zeng C. (2022). PI3K/Akt/mTOR Pathway and Its Role in Cancer Therapeutics: Are We Making Headway?. Front. Oncol..

[20] Janku F. (2017). Phosphoinositide 3-kinase (PI3K) pathway inhibitors in solid tumors: From laboratory to patients. Cancer Treat. Rev..

[21] Wylaź M., Kaczmarska A., Pajor D., Hryniewicki M., Gil D., Dulińska-Litewka J. (2023). Exploring the role of PI3K/AKT/mTOR inhibitors in hormone-related cancers: A focus on breast and prostate cancer. Biomed. Pharmacother..

[22] Schubert K.M., Scheid M.P., Duronio V. (2000). Ceramide Inhibits Protein Kinase B/Akt by Promoting Dephosphorylation of Serine 473. J. Biol. Chem..

[23] Glaviano A., Foo A.S.C., Lam H.Y., Yap K.C.H., Jacot W., Jones R.H., Eng H., Nair M.G., Makvandi P., Geoerger B. (2023). PI3K/AKT/mTOR signaling transduction pathway and targeted therapies in cancer. Mol. Cancer.

[24] Tian J.-M., Ran B., Zhang C.-L., Yan D.-M., Li X.-H. (2018). Estrogen and progesterone promote breast cancer cell proliferation by inducing cyclin G1 expression. Braz. J. Med. Biol. Res..

[25] Ma L., Liu Y., Geng C., Qi X., Jiang J. (2013). Estrogen receptor β inhibits estradiol-induced proliferation and migration of MCF-7 cells through regulation of mitofusin 2. Int. J. Oncol..

[26] Yu X., Zhang X., Dhakal I.B., Beggs M., Kadlubar S., Luo D. (2012). Induction of cell proliferation and survival genes by estradiol-repressed microRNAs in breast cancer cells. BMC Cancer.

[27] Wickramasinghe N.S., Manavalan T.T., Dougherty S.M., Riggs K.A., Li Y., Klinge C.M. (2009). Estradiol downregulates miR-21 expression and increases miR-21 target gene expression in MCF-7 breast cancer cells. Nucleic Acids Res..

[28] Paterni I., Granchi C., Katzenellenbogen J.A., Minutolo F. (2014). Estrogen receptors alpha (ERα) and beta (ERβ): Subtype-selective ligands and clinical potential. Steroids.

[29] Klinge C.M. (2000). Estrogen receptor interaction with co-activators and co-repressors. Steroids.

[30] Shi W.-F., Bartlett J.S. (2008). Estrogen plays a critical role in AAV2-mediated gene transfer in ovarian cancer. Acta Pharmacol. Sin..

[31] Schüler-Toprak S., Moehle C., Skrzypczak M., Ortmann O., Treeck O. (2017). Effect of estrogen receptor β agonists on proliferation and gene expression of ovarian cancer cells. BMC Cancer.

[32] Bateman H.R., Liang Q., Fan D., Rodriguez V., Lessner S.M. (2013). Sparstolonin B Inhibits Pro-Angiogenic Functions and Blocks Cell Cycle Progression in Endothelial Cells. PLoS ONE.

[33] Kim N., Kim C., Ryu S.H., Kim G.O., Bae J.-S. (2022). Anti-Inflammatory Effect of Sparstolonin B through Inhibiting Expression of NF-κB and STAT-1. Int. J. Mol. Sci..

[34] Yang H., Wang B., Wang T., Xu L., He C., Wen H., Yan J., Su H., Zhu X. (2014). Toll-Like Receptor 4 Prompts Human Breast Cancer Cells Invasiveness via Lipopolysaccharide Stimulation and Is Overexpressed in Patients with Lymph Node Metastasis. PLoS ONE.

[35] Lupi L.A., Cucielo M.S., Silveira H.S., Gaiotte L.B., Cesário R.C., Seiva F.R.F., Chuffa L.G.d.A. (2020). The role of Toll-like receptor 4 signaling pathway in ovarian, cervical, and endometrial cancers. Life Sci..

[36] Schilling J.D., Machkovech H.M., He L., Sidhu R., Fujiwara H., Weber K., Ory D.S., Schaffer J.E. (2013). Palmitate and Lipopolysaccharide Trigger Synergistic Ceramide Production in Primary Macrophages. J. Biol. Chem..

[37] Sato Y., Goto Y., Narita N., Hoon D.S. (2009). Cancer Cells Expressing Toll-like Receptors and the Tumor Microenvironment. Cancer Microenviron..

[38] Onier N., Hilpert S., Arnould L., Saint-Giorgio V., Davies J.G., Bauer J., Jeannin J.-F. (1999). Cure of colon cancer metastasis in rats with the new lipid A OM 174. Apoptosis of tumor cells and immunization of rats. Clin. Exp. Metastasis.

[39] Garay R.P., Viens P., Bauer J., Normier G., Bardou M., Jeannin J.-F., Chiavaroli C. (2007). Cancer relapse under chemotherapy: Why TLR2/4 receptor agonists can help. Eur. J. Pharmacol..

[40] Zhang Y.-B., He F.-L., Fang M., Hua T.-F., Hu B.-D., Zhang Z.-H., Cao Q., Liu R.-Y. (2009). Increased expression of Toll-like receptors 4 and 9 in human lung cancer. Mol. Biol. Rep..

[41] Yoo K.H., Lim T.J., Chang S.-G. (2012). Monthly intravesical bacillus Calmette-Guérin maintenance therapy for non-muscle-invasive bladder cancer: 10-year experience in a single institute. Exp. Ther. Med..

[42] Pichika M., Mai C.W., Kang Y.B. (2013). Should a Toll-like receptor 4 (TLR-4) agonist or antagonist be designed to treat cancer? TLR-4: Its expression and effects in the ten most common cancers. OncoTargets Ther..

[43] Li F., Zhang N. (2015). Ceramide: Therapeutic Potential in Combination Therapy for Cancer Treatment. Curr. Drug Metab..

[44] Ogretmen B. (2018). Sphingolipid metabolism in cancer signalling and therapy. Nat. Rev. Cancer.

[45] Yu J.S.L., Cui W. (2016). Proliferation, survival and metabolism: The role of PI3K/AKT/mTOR signalling in pluripotency and cell fate determination. Development.

[46] Sharma V.R., Gupta G.K., Batra N., Sharma D.K., Joshi A., Sharma A.K. (2017). PI3K/Akt/mTOR Intracellular Pathway and Breast Cancer: Factors, Mechanism and Regulation. Curr. Pharm. Des..

[47] Mabuchi S., Kuroda H., Takahashi R., Sasano T. (2015). The PI3K/AKT/mTOR pathway as a therapeutic target in ovarian cancer. Gynecol. Oncol..

[48] Yu L., Wei J., Liu P. (2022). Attacking the PI3K/Akt/mTOR signaling pathway for targeted therapeutic treatment in human cancer. Semin. Cancer Biol..

[49] Afify S.M., Oo A.K.K., Hassan G., Seno A., Seno M. (2021). How can we turn the PI3K/AKT/mTOR pathway down? Insights into inhibition and treatment of cancer. Expert Rev. Anticancer Ther..

[50] Ruvolo P. (2001). Ceramide regulates cellular homeostasis via diverse stress signaling pathways. Leukemia.

[51] Lyu Y., Duan B., Liu Z., Yang F., Chen C., Jiang X., Liu X. (2022). Sparstolonin B inhibits pancreatic adenocarcinoma through the NF-κB signaling pathway. Exp. Cell Res..

[52] Chalfant C.E., Szulc Z., Roddy P., Bielawska A., Hannun Y.A. (2004). The structural requirements for ceramide activation of serine-threonine protein phosphatases. J. Lipid Res..

[53] Matos B., Howl J., Jerónimo C., Fardilha M. (2021). Modulation of serine/threonine-protein phosphatase 1 (PP1) complexes: A promising approach in cancer treatment. Drug Discov. Today.

[54] Aslan M. (2021). Polyunsaturated Fatty Acid and Sphingolipid Measurements by Tandem Mass Spectrometry. Mini-Rev. Org. Chem..

[55] Lee S., Lee D.K. (2018). What is the proper way to apply the multiple comparison test?. Korean J. Anesthesiol..

